# Feasibility and Initial Safety Evaluation of Inhaled ^13^C-Urea in Ambulatory Patients with Pneumonia

**DOI:** 10.1089/jamp.2021.0050

**Published:** 2022-02-14

**Authors:** Justin T. Baca, Nicholas F. Parchim, Jillian Kotulski, Jon Femling, Robert M. Taylor, Silas Bussmann, Lionel Candelaria, Tatsuya Norii, Michele Moyer, Rena Laliberte, Shivan Patel, Samantha Odeesho, Richard Nowak

**Affiliations:** ^1^ Department of Emergency Medicine, The University of New Mexico Health Sciences Center, Albuquerque, New Mexico, USA.; ^2^ Department of Emergency Medicine, Henry Ford Health System, Detroit, Michigan, USA.

Rapid identification of respiratory pathogens is challenging, and point-of-care testing with rapid pathogen identification could improve antibiotic stewardship.^(1)^ Earlier determination of urease-producing organisms, a virulent category of pathogens causing lower respiratory infections, may allow providers to rapidly tailor antibiotic coverage.

**FIG. 1.**
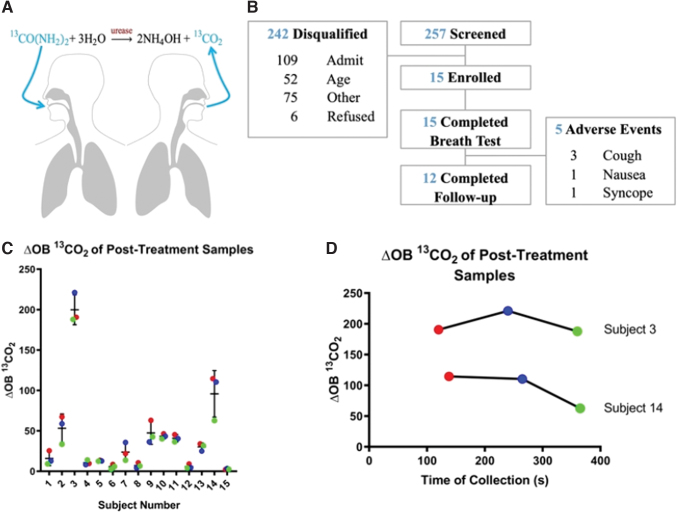
**(A)** Principle of nebulized urea breath testing; **(B)** study overview; **(C)** breath testing results include ΔOB ^13^CO_2_ of post-treatment samples at ∼2 (red), 4 (blue), and 6 (green) minutes collected by direct exhalation. **(D)** Time course for two subjects with the highest ΔOB signal. Fifteen subjects with pneumonia symptomatology and planned for outpatient treatment completed the study. Included subjects were 18–70 years old, capable of providing the BT, and able to understand, agree, and voluntarily consent to participation in the study. In addition, included subjects had no suspected signs or symptoms of bacterial pneumonia, were expected to be treated and then discharged OR a definitive decision to admit had not been made at time of consent, and the subject met all the following criteria: pulse <125 beats per minute, systolic blood pressure ≥100 mmHg, respiratory rate ≤24 breaths per minute, and temperature >35°C and <40°C. Subjects expected to be placed in an ED observation unit were eligible if all the vital sign criteria had been met. Exclusion criteria included a known allergy to urea or any excipient in the nebulized solution, pregnancy or a positive urine pregnancy test result, evidence of active oral infection, known diagnosis of cystic fibrosis or bronchiectasis, known or suspected acute asthma exacerbation upon ED presentation, had received treatment with oral or IV antibiotics in the preceding 48 hours before screening, unless antibiotic failure was suspected OR had received treatment with oral or IV antibiotics >6 hours before BT, or an acute illness or other condition that, as determined by the investigator, would preclude participation in the study. BT, breath test; ED, emergency department; IV, intravenous. Color images are available online.

A breath test (BT) for urease activity was previously described for tuberculosis^(2)^ and pseudomonas colonization in cystic fibrosis.^(3)^ This involves administration of nebulized ^13^C-urea, with increases in exhaled ^13^CO_2_/^12^CO_2_ indicating urea hydrolysis and presence of urease (Fig. 1A). It is unknown whether this approach is feasible in patients with active infection, and the safety of nebulized urea in lower respiratory infection is incompletely characterized.

Subjects presenting to the emergency department (ED) with community-acquired pneumonia, as identified by chest X-ray (CXR) and clinical criteria, and planned for outpatient treatment were approached for participation (Fig. 1B). Subjects received 50 mg nebulized ^13^C-urea dissolved in 3 mL sterile water through an Aerogen Aeroneb Solo nebulizer within 6 hours of antibiotic initiation. We collected breath samples before nebulization and at 2-minute intervals after. Metabolic Solutions (CLIA ID #30D0970292) performed BT analysis. We recorded change in ^13^CO_2_/^12^CO_2_ ratio (delta over baseline or ΔOB, in units of ‰) between pre- and postnebulization samples (Fig. 1C, D). We recorded adverse events (AEs) from enrolment until telephone follow-up at 48–96 hours postnebulization. The Human Research Review Committee of the University of New Mexico Health Sciences Center approved the study (HRRC #16–327) on February 3, 2017, and all subjects provided written consent to participate. Protocol is registered at ClinicalTrials.gov as NCT03100760.

Fifteen subjects, with strong clinical evidence of lower respiratory infection but judged clinically stable for outpatient treatment, completed the study. Subjects were directly observed for at least 20 minutes postnebulization and received standard treatment for pneumonia. Observations were reviewed by the safety monitoring committee.

Three subjects experienced brief coughing, which resolved spontaneously, did not require treatment, and was temporally associated with urea administration. Coughing resolved with a brief interruption of nebulization. There was a single episode of nausea, which was mild and self-limited. One episode of syncope occurred 24 hours after nebulization. The subject had a history of syncope from which the underlying factors remain unclear. Investigators and safety monitor determined these episodes of nausea and syncope to be unrelated to urea administration.

Figure 1C shows ^13^CO_2_ ΔOB for all subjects at 2, 4, and 6 minutes. Subjects are listed in chronological order of enrollment. Subjects 3 and 14 (Fig. 1D) had increased signals and notable clinical indicators of disease. Subject 3 demonstrated bilateral pneumonia on CXR, whereas subject 14 demonstrated infiltrate on CXR with elevated procalcitonin, a marker of possible bacterial infection. Collection of sputum was not mandated in these subjects, planned for outpatient treatment, and no culture results were available.

We believe this to be the largest cohort to date of feasibility and initial safety data for subjects exposed to inhaled isotonic ^13^C-urea during acute lower respiratory infection. This establishes initial logistics of ^13^C-urea BT administration in ambulatory patients presenting to an ED, and who were treated as outpatients for CAP.
